# Multiresistant Bacteria Isolated from Chicken Meat in Austria

**DOI:** 10.3390/ijerph111212582

**Published:** 2014-12-04

**Authors:** Gernot Zarfel, Herbert Galler, Josefa Luxner, Christian Petternel, Franz F. Reinthaler, Doris Haas, Clemens Kittinger, Andrea J. Grisold, Peter Pless, Gebhard Feierl

**Affiliations:** 1 Institute of Hygiene, Microbiology and Environmental Medicine, Medical University of Graz, Graz 8010, Austria; E-Mails: he.galler@medunigraz.at (H.G.); josefa.luxner@medunigraz.at (J.L.); Christian.Petternel@kabeg.at (C.P.); franz.reinthaler@medunigraz.at (F.F.R.); doris.haas@medunigraz.at (D.H.); clemens.kittinger@medunigraz.at (C.K.); andrea.grisold@medunigraz.at (A.J.G.); gebhard.feierl@medunigraz.at (G.F.); 2 Animal Health Service of the Department of Veterinary Administration, Styrian Government, Graz 8010, Austria; E-Mail: peter.pless@stmk.gv.at

**Keywords:** chicken meat, ESBL, *mecA*, *Staphylococcus*, VRE, CTX-M, SHV

## Abstract

Multidrug resistant bacteria (MDR bacteria), such as extended spectrum beta-lactamase (ESBL) *Enterobacteriaceae*, methicillin resistant *Staphylococcus aureus* (MRSA), and vancomycin-resistant Enterococci (VRE), pose a challenge to the human health care system. In recent years, these MDR bacteria have been detected increasingly outside the hospital environment. Also the contamination of food with MDR bacteria, particularly of meat and meat products, is a concern. The aim of the study was to evaluate the occurrence of MDR bacteria in chicken meat on the Austrian market. For this study, 50 chicken meat samples were analysed. All samples originated from chickens slaughtered in Austrian slaughterhouses and were marked as produced in Austria. Samples were analysed for the presence of ESBL *Enterobacteriaceae*, methicillin resistant Staphylococci and VRE. Resistance genes of the isolated bacteria were characterised by PCR and sequencing. In the present study 26 ESBL producing *E. coli,* five *mecA* gene harbouring Staphylococci (but no MRSA), and four VRE were detected in chicken meat samples of Austrian origin. In 24 (48%) of the samples no ESBL *Enterobacteriaceae*, MRSA, methicillin resistant coagulase negative *Staphylococcus* (MRCNS) or VRE could be detected. None of the samples contained all three types of investigated multiresistant bacteria. In concordance to previous studies, CTX-M-1 and SHV-12 were the dominant ESBL genes.

## 1. Introduction

The clinically most relevant MDR bacteria are extended spectrum beta-lactamase (ESBL) *Enterobacteriaceae* and methicillin resistant *Staphylococcus aureus* (MRSA) and also, but less prevalently, vancomycin-resistant Enterococci (VRE). These three groups of resistant bacteria are not only present in the hospital environment, but also in farm animals, meat and meat products [1,2,3,4]. Therefore, the spread of multidrug resistant (MDR) bacteria outside the hospital environment has posed a serious problem over the last few years, and livestock breeding with a rather extensive use of antibiotics has become a possible source for multiresistant bacteria. As a consequence, one possible transmission route for MDR bacteria from animal to human being is food, especially meat and meat products [2,3,5]. 

ESBL *Enterobacteriaceae* are widespread in poultry farming, but were also detected in other farm animals and their meat products [3,6]. Just as with the spread of ESBL in the healthy human population *E. coli* is the most common organism here, but there are differences in strain types and ESBL gene dominance. *E. coli* MLST type ST131 and ESBL enzyme CTX-M-15, the predominant types in humans, are nearly absent in farm animals and food products. In contrast to the observations above, the animal dominant types CTX-M-1, SHV-12 and CTX-M-14 are commonly found in human infections [7,8]. The presence of ESBL genes on mobile genetic elements reduces the impact of the strain background on the spread of ESBL. This fact also complicates the search for resistance reservoirs [7,8,9].

In contrast to ESBL *Enterobacteriaceae*, MRSA show a clearer separation concerning strains of human and animal origin. Livestock-associated (LA)-MRSA are essentially adapted to animals [5,10]. Most of these LA-MRSA lack several virulence factors, like Panton-Valentine leucocidin (PVL) toxin or enterotoxins. Hence serious human infections with LA-MRSA are less frequent than infections caused by hospital acquired (HA)-MRSA or community associated (CA)-MRSA. Studies carried out in Austria reported that up to 8% of all isolated MRSA are LA-MRSA and 10%–20% can be assigned to CA-MRSA. The majority of LA-MRSA detected in humans are considered to be a colonization only (healthy carriers) but also serious infections have been reported [5,11,12,13,14]. The *mec*A gene, which is responsible for methicillin resistance, is located on SCC*mec* cassettes. These are mobile genetic elements responsible for the transfer of resistance genes, but with lower transfer frequency than plasmids or transposons carrying ESBL genes [15,16]. Those gene cassettes can also be part of the genome of coagulase-negative Staphylococci (CNS) species. Several studies indicate that, similar to MRSA, *mec*A harbouring CNS (MRCNS) have a reservoir in animal farming facilities and can also be found in meat products [17,18,19,20].

MRSA and vancomycin-resistant Enterococcus (VRE) are endemic in hospital settings and long-term care facilities, and the prevalence of colonization is increasing [21,22]. Enterococci are intestinal bacteria which colonize mammals, birds, reptiles and insects. Currently, nine different variants of vancomycin resistance genes have been described in Enterococci (*van*A, B, C, D, E, G, L, M and N). *Van*A and *van*B are the most clinically relevant types leading to many cases of colonization but only sporadic infections in hospitals. VRE are among the first documented antibiotic resistant bacteria with primary origin in animal farming. The rise of VRE was caused by the use of the glycopeptide avoparcin as a growth promoter, starting from 1975. After that it was used extensively, mainly in broiler and pig farming. But, as avoparcin confers cross resistance to vancomycin, the (mis)use of avoparcin resulted in the selection of VRE until it was finally banned in animal farming in the whole European Union in 1996. A study carried out in Denmark reported a decline from 80% to 5% in the occurrence of VRE isolated from broilers in the period from 1995 to 1998. In Central Europe the presence of VRE isolated from human patients is stable at the level of 1% since 2000. Several studies show local rates up to 15%. This seems to be more related to nosocomial outbreaks [23,24,25,26,27,28]. The aim of this study was to investigate the presence of ESBL *Enterobacteriaceae*, methicillin resistant Staphylococci (MRSA and MRCNS) and VRE in chicken meat products slaughtered in Austria and the determination of (genetic) characteristics of the isolated strains. Samples were taken from the four main supermarket chains and from local butchers, in order to get an impression of chicken meat contamination with MDR bacteria.

## 2. Material and Methods

### 2.1. Samples

In the course of this study 50 samples of chicken meat (chm-sample-1 to chm-sample-50) were analysed. All samples originated from chickens slaughtered in Austrian slaughterhouses and were marked as produced in Austria. From January to March 2012, fresh meat samples (without skin) from four different supermarket chains as well as butchers were bought in the city of Graz. The samples were transported to the laboratory in a cooling-box at 4 °C and the investigation process was started immediately after arrival in the laboratory.

### 2.2. Strain Isolation and Detection

The preparation of the food samples was done according to ISO 6887-2:2003 [29]. Two enrichment methods were chosen for ESBL and MRSA: the first with peptone broth, the second with thioglycolate bouillon. VRE enrichment was done with enterococcosel (Becton Dickinson Austria, Vienna, Austria). 25 g of the meat samples were randomly selected, mixed with 225 mL peptone broth 1% (Oxoid Ltd, Basingstoke, England) and homogenized in a Stomacher^®^ 400 Lab Blender for two minutes. The solution was poured into an Erlenmeyer flask and enriched overnight (16 to 24 hours) in an incubator shaker (Innova 4000, New Brunswick Scientific, Enfield, USA) at 150 rpm at 37 °C. After that, a decimal dilution series from peptone broth and enterococcosel enrichment up to 10^−3^ was performed. 0.1mL from the appropriate dilution was inoculated on chromeID^TM^ ESBL (bioMérieux, Marcy-l’Etoile, France), chromeID^TM^ VRE (bioMérieux) and oxacillin resistance screening agar (OXA agar, Oxoid Ltd.). ChromeID^TM^ ESBL agar was incubated for 24 hours at 37 °C; chromeID^TM^ VRE and OXA agar for 48 h at 37 °C. Colonies were assessed as described in the manufacturer’s manual and identified with Vitek MS (bioMerieux) [30]. In the second enrichment method, 1 g of each meat sample was inoculated in 5 mL thioglycolate bouillon and incubated overnight (16 to 24 h) at 37 °C. A sterile cotton swab was dipped into the thioglycolate bouillon and inoculated on the selective agar plates mentioned above. Bacteria were identified as described above. ESBL-positive *E. coli* were confirmed by CLSI Screening and Confirmatory Tests [31]. Resistance to the glycopeptides vancomycin and teicoplanin was confirmed by Etest (bioMérieux) according to the manufacturer’s instructions.

### 2.3. Antimicrobial Susceptibility Testing

For all identified *Enterobacteriaceae*, *Staphylococcus* spp and *Enterococcus* spp susceptibility testing was performed as recommended by the European Committee on Antimicrobial Susceptibility testing (EUCAST). The inhibition zone diameters were interpreted according to EUCAST guidelines, except Enterobacteriaceae tested for tetracycline, chloramphenicol and nalidixic acid, which were evaluated in conformity with Clinical Laboratory Standards Institute (CLSI) guidelines. There a no interpretation guidelines for zone diameters of these three antibiotics according to EUCAST (23, 24).

For *Enterobacteriaceae* ampicillin (10 µg), amoxicillin/clavulanic acid (20 µg/10 µg), piperacillin/tazobactam (100 µg/10 µg), cefalexin (30 µg), cefuroxime (30 µg), cefoxitin (30 µg), cefotaxime (5 µg), ceftazidime (10 µg), cefepime (30 µg), gentamicin (10 µg), trimethoprim/sulfamethoxazole (1.25 µg/23.75 µg), ciprofloxacin (5 µg), moxifloxacin (5 µg), imipenem (10 µg), meropenem (10 µg), tetracycline (30 µg), nalidixic acid (30 µg) and chloramphenicol (30 µg) BD BBL^TM^, Sensi-Disc^TM^ paper discs (Becton, Dickinson and Company, Sparks, MD, USA) were used. Antimicrobial susceptibility for *Staphylococcus* spp was tested with penicillin (1 µg), cefoxitin (30 µg), tetracycline (30 µg), erythromycin (15 µg), clindamycin (2 µg), norfloxacin (10 µg), gentamicin (10 µg), trimethoprim/sulfamethoxazole (1.25 µg/23.75 µg), fusidic acid (10 µg), rifampicin (5 µg), linezolid (10 µg) and mupirocin (200 µg) BD BBL^TM^ Sensi-Disc^TM^ paper discs. Antimicrobial susceptibility for *Enterococcus* spp was determined for ampicillin (2 µg), vancomycin (5 µg), teicoplanin (30 µg), linezolid (10 µg) and tigecycline (15 µg) by disc diffusion test, using BD BBL^TM^ Sensi-Disc^TM^ paper discs, according the EUCAST guidelines (V2.0 2012). 

### 2.4. Detection of Resistance Genes

PCR detection and gene identification were carried out for three different β-lactamase gene families, *bla*_TEM_, *bla*_SHV_ and *bla*_CTX-M_ (including subgroup 1, 2 and 9). PCR and sequencing procedures were performed as described previously [32,33]. Conditions for PCR: initial denaturation at 94 °C for 5 min; 35 cycles at 95 °C for 30 s, 52 °C for 45 s, and 72 °C for 60 s; and final incubation for 10 min at 72 °C. Taq DNA polymerase and dNTPs from QIAGEN (Hilden, Germany) were used. Sequences were compared to NCBI database. The detection of the *mec*A genes was performed as described previously [34] and the detection of the vancomycin resistance genes (*van*A/*van*B*)* was performed by real time PCR with the Light cycler VRE Detection Kit (Roche, Branchburg, New Jersey, USA) [35].

## 3. Results

### 3.1. ESBL Enterobacteriaceae

In 21 samples 26 different ESBL *E. coli* were detected; no other ESBL positive *Enterobacteriaceae* species were recovered. In five samples two phenotypically and genotypically different ESBL *E. coli* could be isolated. Three different ESBL genes were responsible for (ESBL) resistance in these isolates: Twelve isolates had genes encoding for CTX-M-1 enzyme, twelve for SHV-12 and two for SHV-2. The gene for non ESBL TEM-1 enzyme was also detected in three strains (two times in combination with CTX-M-1 and one time with SHV-12) (Table 1). 

**Table 1:** Resistance genes and antibiotic resistance profile of multiresistant bacteria isolated from chicken meat.

Isolate	Species	Resistance Gene	Sample	Resistance Pattern
ESBL-1	*E. coli*	*bla*_SHV-12_	chm-sample-1	caz, tet, na, c
ESBL-2	*E. coli*	*bla*_CTX-m-1_	chm-sample-1	fep, tet, na, c
ESBL-3	*E. coli*	*bla*_SHV-12_	chm-sample-2	caz, fox, sxt, tet,na,c
ESBL-4	*E. coli*	*bla*_CTX-m-1_	chm-sample-3	fep, tet,na
ESBL-5	*E. coli*	*bla*_CTX-m-1_	chm-sample-9	tet,na
ESBL-6	*E. coli*	*bla*_SHV-12_	chm-sample-9	caz, tet,c
ESBL-7	*E. coli*	*bla*_CTX-m-1_	chm-sample-10	fep, tet
ESBL-8	*E. coli*	*bla*_SHV-12_	chm-sample-16	caz, tet, c
ESBL-9	*E. coli*	*bla*_CTX-m-1_, *bla*_TEM-1_	chm-sample-17	fep, sxt, tet, c
ESBL-10	*E. coli*	*bla*_SHV-12_	chm-sample-19	caz, tet, na, c
ESBL-11	*E. coli*	*bla*_CTX-m-1_	chm-sample-21	fep, fox, tet, na, c
ESBL-12	*E. coli*	*bla*_SHV-12_, *bla*_TEM-1_	chm-sample-21	caz, mxf, tet, na, c
ESBL-13	*E. coli*	*bla*_SHV-12_	chm-sample-22	caz, tet, na, c
ESBL-14	*E. coli*	*bla*_CTX-m-1_	chm-sample-25	tet, na
ESBL-15	*E. coli*	*bla*_SHV-12_	chm-sample-28	caz, tet, na, c
ESBL-16	*E. coli*	*bla*_SHV-2_	chm-sample-33	na
ESBL-17	*E. coli*	*bla*_CTX-m-1_	chm-sample-34	fep, tet
ESBL-18	*E. coli*	*bla*_CTX-m-1_	chm-sample-36	fep, tet
ESBL-19	*E. coli*	*bla*_CTX-m-1_	chm-sample-39	fep, sxt, tet, na
ESBL-20	*E. coli*	*bla*_CTX-m-1_, *bla*_TEM-1_	chm-sample-39	tet
ESBL-21	*E. coli*	*bla*_SHV-2_	chm-sample-40	sxt, tet, na
ESBL-22	*E. coli*	*bla*_SHV-12_	chm-sample-43	caz, tet, na
ESBL-23	*E. coli*	*bla*_CTX-m-1_	chm-sample-44	fep, na
ESBL-24	*E. coli*	*bla*_SHV-12_	chm-sample-44	caz
ESBL-25	*E. coli*	*bla*_SHV-12_	chm-sample-45	caz, tet, na, c
ESBL-26	*E. coli*	*bla*_SHV-12_	chm-sample-50	caz, tet, na
VRE-1	*E. faecium*	*van*A	chm-sample-17	am, tec
VRE-2	*E. faecium*	*van*A	chm-sample-23	tec
VRE-3	*E. faecium*	*van*A	chm-sample-33	am, tec
VRE-4	*E. faecium*	*van*A*, van*B	chm-sample-33	am
MRCNS-1	*Staph. fleurettii*	*mec*A	chm-sample-9	
MRCNS-2	*Staph. sciuri*	*mec*A	chm-sample-11	
MRCNS-3	*Staph. sciuri*	*mec*A	chm-sample-16	
MRCNS-4	*Staph. vitulinus*	*mec*A	chm-sample-18	
MRCNS-5	*Staph. vitulinus*	*mec*A	chm-sample-49	

All isolates were susceptible to the tested aminoglycosides, β-lactam/β-lactamase inhibitor, carbapenems, ciprofloxacin and tigecycline. Highest resistance rates were observed for tetracycline in 23 (88.46%) of the isolates and nalidixic acid in 18 (78.26%) isolates. All isolates were resistant against the third generation cephalosporine cefotaxime. In addition, the SHV-12 positive isolates (46.15% of isolated ESBL *E. coli*) showed non-susceptibility to third generation ceftazidime (10 µg). Nine isolates (34.61%), all positive for the CTX-M-1 gene, were resistant against the fourth generation cephalosporin cefepime (Table 1).

### 3.2. MRSA and MRCNS

All samples were tested negative for MRSA. However, five coagulase negative *Staphylococcus* strains, recovered from MRSA screening Agar, were positive for the *mecA* gene (10 % of the samples). They were identified as *Staphylococcus vitulinus* (two), *Staphylococcus sciuri* (two) and *Staphylococcus fleurettii* (one) (Table 1). Beside resistance to penicillin and cefoxitin, none of these strains showed additional resistance.

### 3.3. VRE

VRE were found in three of 50 samples (6%). A total of four *Enterococcus* strains, different in their *van* genes, were recovered and analysed in detail. All isolates were identified as *Enterococcus faecium* and harboured the *van*A gen whereas one isolate (VRE-4) was additionally *van*B positive. All isolates showed highly similar resistance patterns. They were all resistant to vancomycin, and susceptible to tigecycline and linezolid. Only one isolate was susceptible to teicoplanin (VRE-4) and one to amoxicillin (VRE-2) (Table 1). 

## 4. Discussion

Besides the international trade of farm animals, intensive antibiotic use is an important factor for the spread of livestock-associated, multiresistant bacteria. Presence of *E. coli* ESBL in poultry and poultry meat products is documented by many studies from all over the world. High contamination rates with ESBL Enterobacteriaceae, up to 93.3%, are reported [6,36,37]. Austria and surrounding countries do not seem to observe such high rates of MDR bacteria in meat although in some cases such as in Austria, data are extremely limited. The study from 2012 Springer and Bruckner with samples from 2009 revealed a little lower ESBL frequency (35.9% of chicken meat samples) but with a very low SHV frequency [38]. In contrast, recent German studies by Campos *et al.* [39] and Kola *et al.* [40] detected SHV-12 as second or most common ESBL enzymes in poultry meat. Frequencies of total ESBL *E. coli* in these two studies were similar (Kola *et al.* 43.9% samples from 2011) or higher (Campos *et al.* 60% samples from 2012).

Therefore the results of the present study concerning SHV-12 and CTX-M-1 are in concordance with those of other European countries. Both genes are very common in animal farming. SHV-12 is known to be associated mainly with poultry and CTX-M-1 is widespread in mammals. In contrast to other studies, TEM-52 and CTX-M-14, also common in farm animals and meat products, were not detected here [36,37,39,40]. Two reasons for the absence of these genes might be the relatively small sample size and the exclusive Austrian origin of the poultry.

In concordance with previous studies, the resistance against tetracycline is shown to be common in farm animals and meat product samples because tetracycline is known to be still in use in livestock breeding. In contrast to this, in the present study fluoroquinolone resistance was nearly absent, although fluoroquinolones are frequently used antibiotics in human healthcare in Austria. This finding can not only be explained with the connection of this resistance to the occurrence of CTX-M-15 because reports from non-CTX-M-15 ESBL *E. coli* from Austria show that fluoroquinolone resistance is present in human and also environmental isolates [7,38,41,42,43]. 

LA-MRSA are known to be associated essentially with mammalian farm animals but was also isolated from poultry and poultry meat products. Contamination of meat with HA- or CA-MRSA, possibly caused due to the lack of hygiene during meat production, is also documented. Hence the absence of MRSA in this study was surprising [44,45,46]. But the *mec*A gene was present in coagulase negative Staphylococci in animal farming years before the first LA-MRSA was reported. The detected MRCNS species in this study are also in concordance with previous studies [47,48,49]. Although avoparcin was banned in animal feeding in the European Union more than two decades ago, VRE are still present in the meat production. The persistence of VRE over a prolonged time period is potentially caused by co-selection with other food additives [24,25,50].

## 5. Conclusions

ESBL *E. coli* was the most common type of multiresistant bacteria observed in this study. Testing revealed 21 samples out of 50 (42%) as positive for ESBL. No MRSA could be detected but five samples were positive for MRCNS. VRE could be recovered from three samples (6%). None of the samples contained all three types of multiresistant bacteria, but three samples harboured *E. coli* ESBL together with MRCNS, and in two samples *E. coli* ESBL emerged additionally to VRE. In 24 (48%) samples no ESBL Enterobacteriaceae, MRSA, MRCNS or VRE could be detected. This study had a very limited sample size, and thus limited power to infer the prevalence of antimicrobial resistance in chicken meat in Austria. Although informative as a starting point, especially considering the lack of Austrian data, further work on this matter in Austria should occur.
